# The influence of two different cements on remaining cement excess in cement-retained implant-supported zirconia crowns. An in vitro study

**DOI:** 10.1038/s41405-021-00063-8

**Published:** 2021-01-28

**Authors:** Jazmin Hidalgo, Desirée Baghernejad, Anders Falk, Christel Larsson

**Affiliations:** 1 Master student, Department of Materials Science and Technology, Faculty of Odontology, Malmö University, Malmö, Sweden; 2 Senior lecturer, Department of Materials Science and Technology, Faculty of Odontology, Malmö University, Malmö, Sweden; 3 Associate professor, Department of Prosthodontics, Faculty of Odontology, Malmö University, Malmö, Sweden

**Keywords:** Calcium-based cement, Glass-ionomer cement, Fixed prosthodontics, Dental implants

## Abstract

**Aim:**

To compare the amount of remaining cement excess after cementation of implant-supported zirconia crowns with zinc phosphate or calcium aluminate glass ionomer cement.

**MATERIALS AND METHODS:**

Twenty zirconia crowns were cemented on dental implant abutments using a calcium aluminate glass ionomer cement (*n* = 10) and zinc phosphate cement (*n* = 10). After removal of cement excess, remaining cement excess were measured with pixel area calculation method and by weighing. Differences in amount of remaining cement excess were analyzed using Independent Samples *t*-Test. Level of significance was set at *p* = 0.05.

**Results:**

Zinc phosphate cement had a significantly greater amount of remaining cement excess than calcium aluminate glass ionomer cement in terms of total number of pixels (*p* = 0.002) and amount in grams (*p* = 0.005).

**Conclusion:**

The study suggests that the amount of remaining cement excess can be affected by the type of cement. Calcium aluminate glass ionomer cement may be a more suitable choice for cement-retained dental implant restorations, and possibly reduce the risk of complications related to cement excess such as peri-implant disease. Further studies are needed to verify the results from the present study.

## Introduction

Dental implants have become a widely used treatment for the rehabilitation of partially and completely edentulous patients.^1,2^ Implant-supported dental restorations can be retained through screw- or cement-retention.^3^ A considerable disadvantage with cement retention is the possible biological complications.^3,4^ Remains of cement excess can irritate surrounding peri-implant tissue in a manner similar to subgingival tartar.^4^ In addition, bacteria can adhere to the rough surface of the remaining cement excess and cause inflammation.^5–7^ These processes have been suggested as major^7^ etiological factor behind peri-implant disease.^8^ Peri-implant disease is a multifactorial process where factors such as smoking, poor oral hygiene, genetics and history of periodontal infection are predisposing risk factors, but there is no general agreement regarding which factor is more consequential.^8^ According to Wittneben et al.^9^ biological complications such as fistulas and suppuration, are more frequently found among cement-retained dental implant restorations than screw-retained ones. Moreover, Sailer et al.^10^ observed bone loss over 2 mm more frequently around cement-retained dental implant restorations in comparison with screw-retained dental implant restorations. Wilson et al.^7^ evaluated dental implants with signs of peri-implant disease. Remaining cement excess was associated with 81% of the affected implants.^7^ In addition, signs of inflammation disappeared when cement excess were eliminated.^7^ These findings suggest a potential significant detrimental effect of cement excess on the peri-implant tissues.^7^ Other groups such as Korsch et al.^11^ and Linkevicius et al.^8^ have found similar results.

Previous studies have shown that there are difficulties in removing cement excess. The removal process of the cement excess is particularly difficult when the finish line of the preparation is below the mucosal margin.^12,13^ Other factors such as undercuts, low cement viscosity^5^ and wider implant diameter^14^ can also result in a greater amount of remaining cement excess.^5^ It is possible that the type of cement may influence the process of cement removal. A water-based hybrid glass ionomer cement, composed of calcium aluminate and glass ionomer (CAGIC),^15^ is said to have a rubber-like consistency during setting. This property may allow for easier removal than conventional cements such as zinc phosphate (ZNPH). CAGIC is intended for cementation of fixed dental restorations and fulfils the requirements of the ISO standard (9917:1[2003]).^16^ The short setting time and strength are obtained from the glass ionomer components.^15^ The mean setting time for CAGIC is 4.8 ± 0.1 min,^16^ which is reduced compared to ZNPH (5.5 min), glass ionomer (7.0 min) and increased compared to resin cement (2.0–4.0 min).^15^ The material has good flowability which gives a thin film thickness, 16.8 ± 0.9 μm,^16^ which is thinner than ZNPH (20 μm), glass ionomer (24 μm) and resin cement (<25 μm).^15^ The calcium aluminate contributes to reduced microleakage, adequate biocompatibility and long-term strength and stability.^15^ Compressive strength for CAGIC is ~160 MPa and thereby higher than the ISO requirements for water-based cements (50 MPa) and higher than ZNPH and glass ionomer cement (104 MPa respectively 86 MPa).^15,16^ Hardness is higher for CAGIC (VHN 68.3 ± 17.2) than ZNPH (VHN 51.4 ± 10), which could influence ease of removal.^16^

Few studies have compared how effectively cement excess can be removed comparing different types of cement. Therefore, the aim of this study was to compare the amount of remaining cement excess after cementation of implant-supported zirconia crowns with CAGIC and ZNPH cement. The hypothesis was that cementation with CAGIC will leave less remaining cement excess than ZNPH cement, due to CAGIC’s handling properties.

## Methods

### Preparation of specimens

**Fig. 1:**
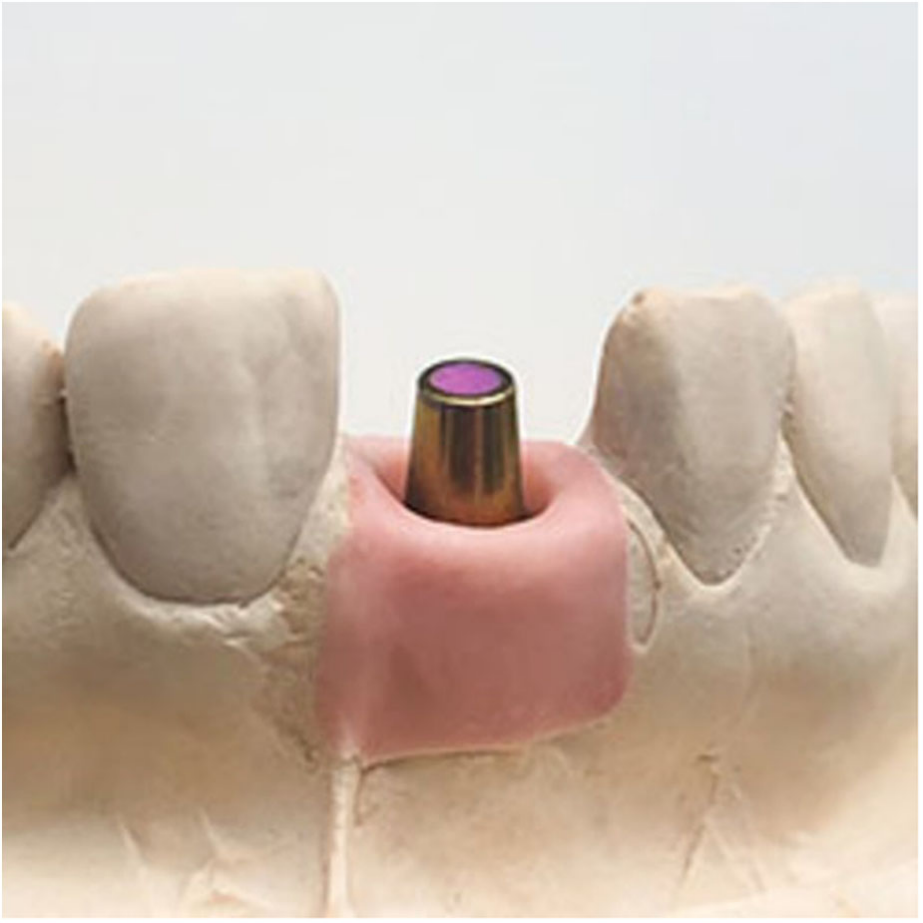
Dental cast. Dental cast with gingival mask and dental abutment in the position of the maxillary right central incisor.

A dental technician produced 22 identical dental casts (Mircomod type IV, Zeuz srl., Italy) which were provided with implant analogs (Lab Analog [AR]⌀4~⌀5.5/L = 12, MegaGen Implant Co. Ltd., Republic of Korea) in the position of the maxillary right central incisor. Twenty-two identical gingival masks (Zhermack Gingifast rigid, Zhermack S.p.A., Italy) were produced. Each implant analog was provided with an abutment (EZ Post Abutment [AR]⌀5/ C = 2/ P = 5.5/Hex, MegaGen Implant Co. Ltd., Republic of Korea) with a guide chute placed distally and a finish line placed ~1.5 mm below the gingival margin (Fig. 1).

Twenty-two crowns were designed in dental wax (StarWax CB, Dentaurum GmbH & Co. KG, Germany), scanned (Straumann® 3Series, Dental Wings inc., Canada) and thereafter milled (CORiTEC 350i, imes-icore GmbH, Germany) in zirconia (DD cube ONE® – High Translucent Plus (HT+), Dental Direkt, Germany) at a milling center (Implant Solutions West AB Sweden). The crowns were milled with a cement gap of 0.35 μm. Before choosing 0.35 μm cement gap, various test crowns with different cement gaps ranging from 0 to 0.5 μm were tested in a pilot trial. Two models were used in this pilot. Three tests were performed, in the first test, an a-silicone impression material was used to replicate the interfaces between the crowns and the abutments finish line to check if each respective gap size allowed correct seating, where the crowns reached the intended finish lines. In the second test, test crowns were cemented with CAGIC (Ceramir® Crown & Bridge, Doxa Dental AB, Sweden) and ZNPH (Harvard Cement, Harvard Dental International GmbH, Germany). The finish lines were probed to check proper seating of the crowns onto the abutments. In the third test, all crowns were set with a passive fit on the abutments and rotated by hand mesially and distally around the abutment’s axis of rotation. Thereafter, two crowns with the cement gap of 0.35 μm were cemented onto the two models used in the pilot trial, to test the method of this study. The remaining 20 crowns and dental casts were divided into two groups; 10 crowns cemented onto 10 titanium abutments with CAGIC in one group (test) and 10 crowns cemented onto 10 titanium abutments with ZNPH in the other (control).

**Fig. 2:**
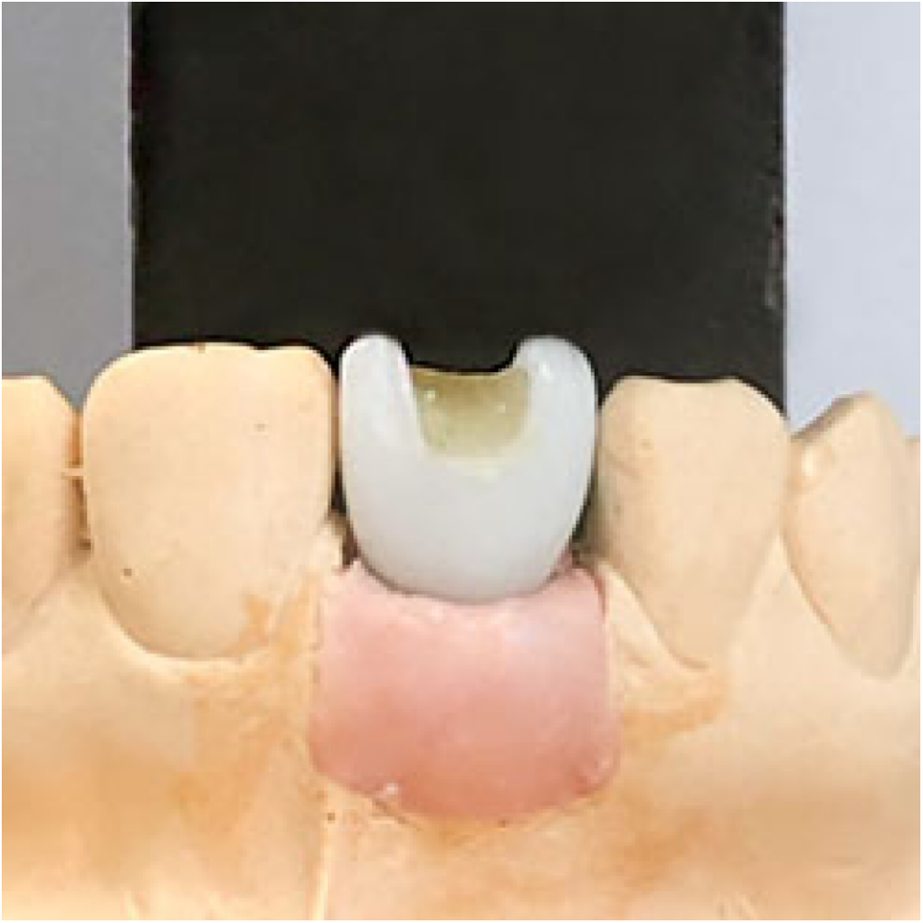
Composite lid. Zirconia crown with composite covered venting holes.

Before cementation, teflon tape (PTFE sealing tape, NASTRO, Italy) and a-silicone impression material (3M™ ESPE™ Imprint™ 4 Light, 3M ESPE, Germany) were applied in the screw access holes to protect the abutment screws during cementation. The crowns were designed with venting holes to facilitate the removal of the crown and the abutment in one piece, from now on called specimen, after cementation. The venting holes were covered with a composite lid (Filtek™ Supreme XTE, 3M ESPE, Germany) to prevent any leakage of cement during cementation (Fig. 2).

**Fig. 3:**
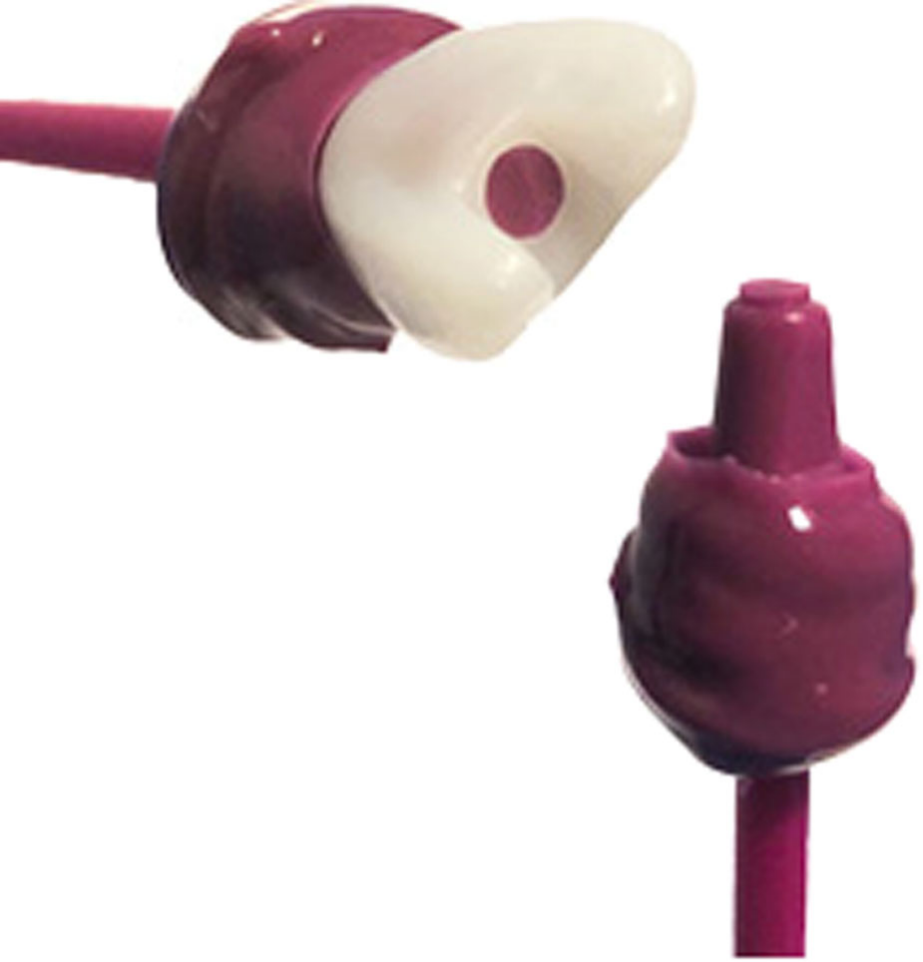
Silicone replica. Silicone replica with and without zirconia crown.

To obtain a comparable and even inner surface of composite on all crowns, one crown was overfilled with a-silicone and allowed to set. Thereafter the occlusal excess silicone was removed with a scalpel (Surgical Scalpel Handle Number 3GS/S and blade No.11, Swann-Morton®, England) and the remaining silicone inside the crown formed a silicone replica that was used on all crowns to prevent composite from interfering with the cement gap (Fig. 3). The occlusal surfaces adjacent to the venting holes of the crowns were coated with adhesive (Scotchbond™ Universal Adhesive, 3M ESPE, Germany) using a quick stick (Quick Stick, Dentsolv AB, Sweden) and thereafter cured with a dental curing light (Translux® Wave, Heraeus Kulzer GmbH, Germany) for 10 s. The composite was applied with a composite instrument (LM-ErgoMax handle LM 48–702 XSI, © LM-Instruments Oy, Finland) and hardened for 20 s with a dental curing light. The abutments and crowns were rinsed with ethanol (Etanol APL Dentallösning 99.5 % v/v, APL Pharma Specials, Sweden) and water to remove any type of contamination and thereafter gently dried.

### Cementation

The zirconia crowns, covered with composite lids, were weighed separately on a scale with an accuracy of 0.0001 g (Adventurer Pro AV264, Ohaus®, OHAUS Europe GmbH, Switzerland) before cementation in order to be able to determine the weight of the cement at a later stage. Cement capsules were activated according to the manufacturer’s recommendations. Immediately after activation, the CAGIC and ZNPH capsules were mixed for 8 and 10 s respectively with a high-frequency (4500 rpm) oscillating mixer (Silamat® S5, Ivoclar Vivadent AG, Austria). The time was recorded from end of mixing until the cement had been set. The capsules were inserted into an applicator (Aplicap™ System Capsule Applier, 3M ESPE, Germany) and the cement was pressed out and filled approximately one third of the crowns. With the help of a brush (Top Dent penselborste and Penselborste Normallång, transparent, TOP DENT, Sweden), the cement was applied on the intaglio surface of the crowns. The crowns were thereafter weighed to achieve approximately the same amount of cement in all crowns and to determine the weight of the cement that was used for each crown. After 1 min, the crowns were placed on the abutments with maximal finger pressure of approximately 55 N for 3 min for CAGIC and 10 min for ZNPH. The time recommended by the manufacturer for removal of cement excess, was doubled as a precaution as cementation was performed outside the mouth. After 4 min CAGIC reached a rubber-like consistency and at this stage the cement excess was removed with a straight stainless-steel probe (Sond Rak, Depro AB, Sweden) and dental floss (Tandtråd, Lifco Dental AB, Sweden) to imitate the clinical situation. Thereafter an axial load of 52 N was applied with a loading device with prefabricated controlled weights for 5 min. After applying a maximal finger pressure for 10 min, ZNPH was also subjected to a load of 52 N with the loading device for 5 min. The cement excess was also removed with a probe and dental floss. The cement excess removal process was completed when the operator perceived that the cement excess had been completely removed. After 24 h, when the cements were fully hardened, a hole was drilled through the composite lid to access the abutment screw and the specimen was removed from the dental cast using a screwdriver (Abutment Removal Driver M1.8/Long, MegaGen Implant Co. Ltd., Republic of Korea). To avoid inter-observer variations, each step was carried out by the same operator.

### Measurement of remaining cement excess

**Fig. 4:**
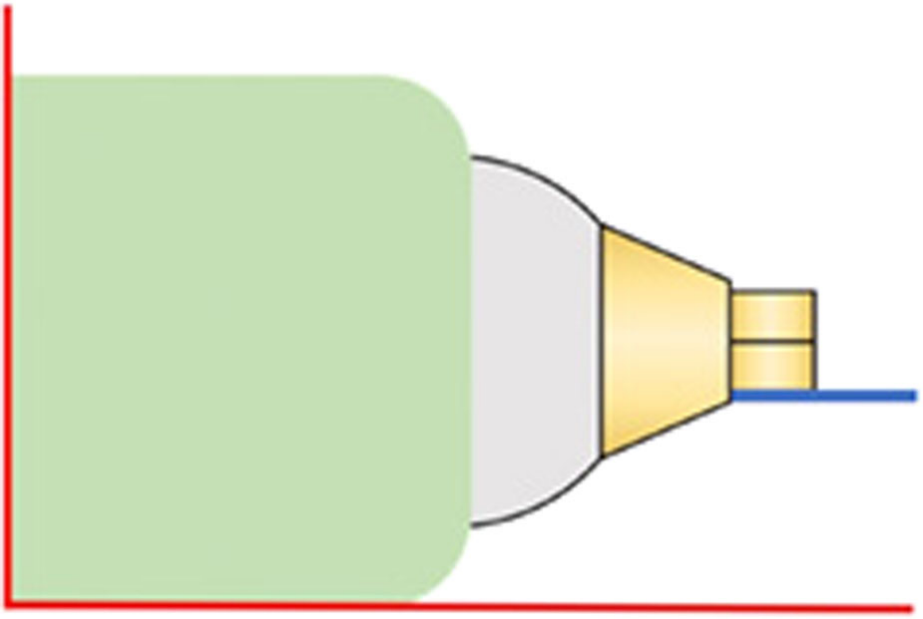
Specimen in putty mold (green), side view. Red lines representing flat surfaces at right angle to each other, blue line representing one hexagonal side parallel to flat surface (red line).

**Fig. 5:**
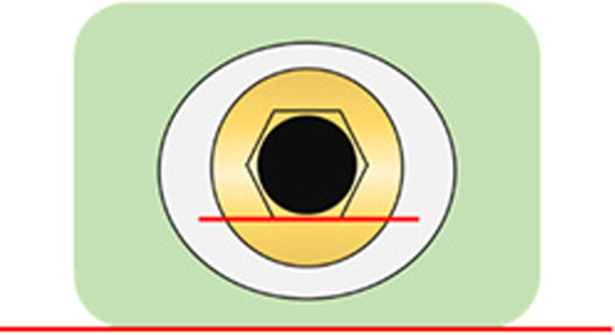
Specimen in putty mold (green), axial view. Red lines representing flat surface parallel to one hexagonal side.

**Fig. 6:**
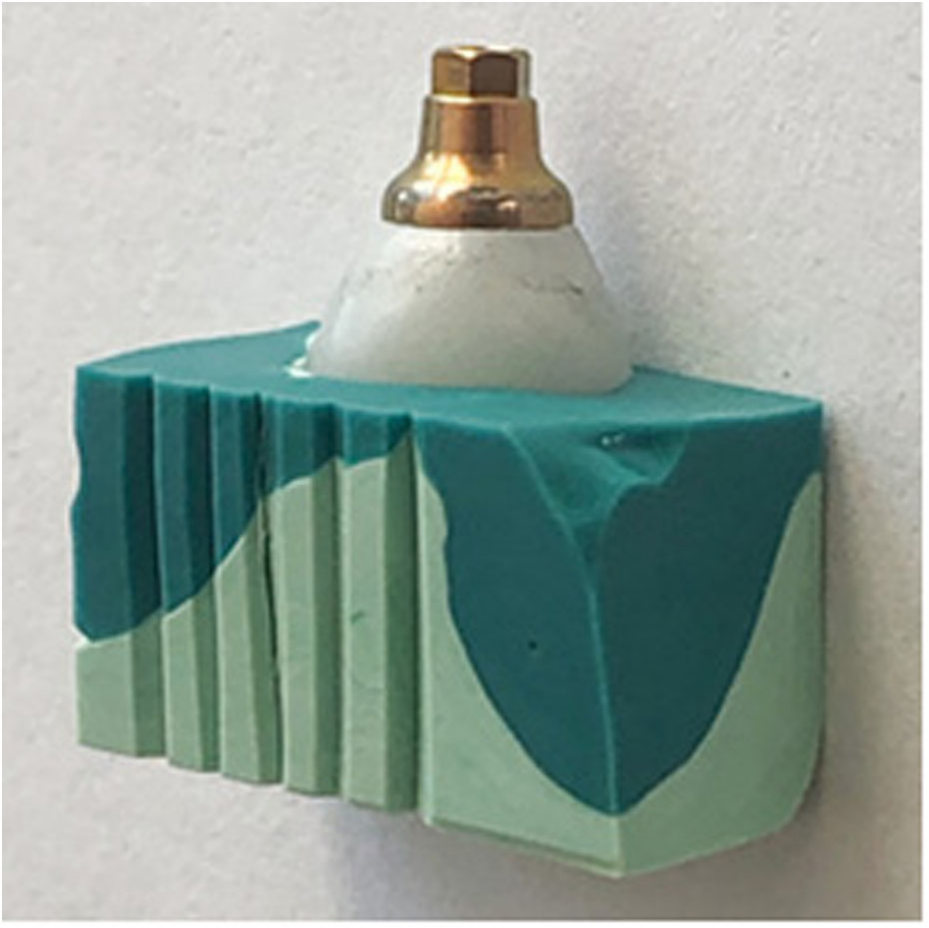
Putty mold with specimen. Putty (light green) and a-silicone impression material (dark green).

Six molds of dental impression putty (Provil® novo Putty regular set, Kulzer GmbH, Germany) and a-silicone impression material (Provil® novo Light fast set, Heraeus Kulzer GmbH, Romania) were made to fixate the specimen in six different positions to facilitate measurements of the remaining cement excess. The molds were made based on each hexagonal side of the abutment’s internal connection. A small amount of putty was mixed according to the manufacturers’ recommendations and placed on two flat surfaces at a right angle to each other (Fig. 4). The specimens were fixed in the putty with one of the hexagonal sides aligned with the flat surface (Fig. 5). After the putty was set, silicone was applied over the putty and around the specimen to form a more flexible and reusable mold (Fig. 6).

**Fig. 7:**
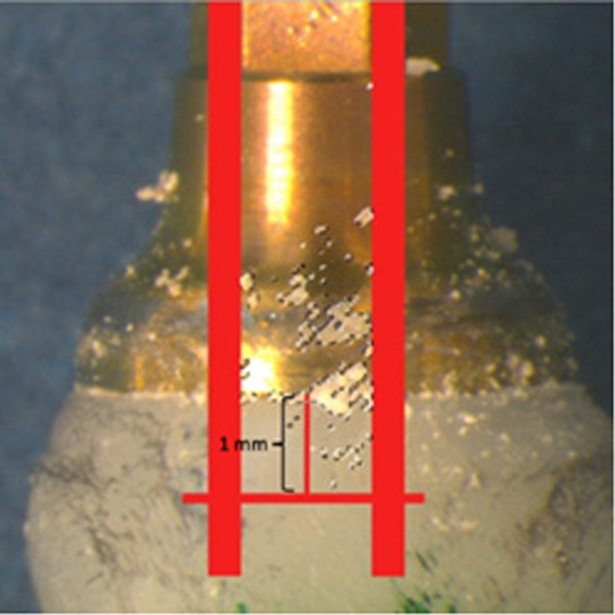
Measurement tool. Highlighted pixels of cement excess within the measurement tool (red).

After the specimens were removed from the casts, the remaining cement excess was measured using two methods, pixel area calculation and weighing. Each specimen was inserted in all six putty molds (Fig. 6) and photographed with a digital camera (Leica DFC420, Leica Microsystems Ltd., Germany) connected to a microscope (Wild M7A Zoom stereomicroscope, Wild Heerbrugg, Switzerland). The pictures were processed in a computer program called Leica Suite (Leica Application Suite, Version 4.1.0 [Build: 1264], Copyright© 2013–2012, Leica Microsystems CMS GmbH, Switzerland). The microscope was first calibrated and then set at 12× zoom. One hexagonal side of the abutment represented one area of measurement, resulting in six measurements per specimen, with a total sample size of 120. Pictures taken with the microscope were imported to Photoshop (Adobe® Photoshop® CS5 Extended Version 12.0.4 ×64, Adobe Systems Incorporated, USA) and a tool to limit the area of measurement was created. The tool consisted of two parallel vertical lines which were drawn along the outer limitations of one hexagonal side and one horizontal line which was drawn 1 mm coronally beyond the finish line. These lines formed a boundary where only the cement excess within the lines was measured (Fig. 7).

With the selection brush tool in Photoshop, the cement excess in the pictures was manually marked. Cement excess smaller than pixel size 8 were not measured due to difficulties in determining the border of the cement excess. The total amount of marked pixels was obtained with the histogram function. Thereafter, the cement excess on the specimens and in the gingival masks were removed with a probe and weighed.

### Statistical analysis

The sample size was determined by comparison to other studies, such as Chee et al.^6^ and Linkevicius et al.^13^ The results were analyzed with SPSS version 25 (IBM SPSS Statistics for Windows, IBM Corp., USA) with the help of a statistician. Mean values and standard deviation were calculated. Thereafter, an Independent Samples *t-*Test was carried out in SPSS version 25 to investigate if there was a statistically significant difference between the two groups, in terms of weight and number of pixels. Level of significance was set at *p* = 0.05.

## Results

**Fig. 8:**
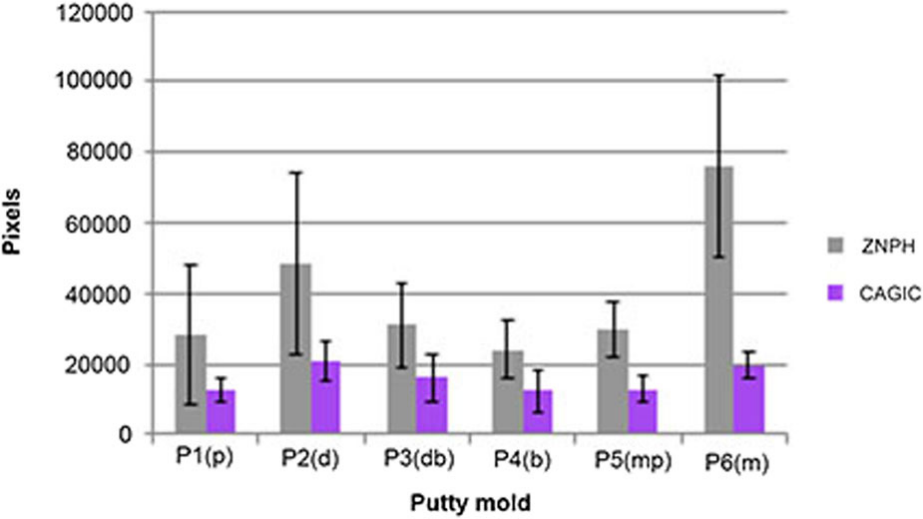
Results of pixel area calculation. Mean of total number of pixels of remaining cement excess for ZNPH and CAGIC, with 95% confidence interval. P1–P6 representing each putty mold with surfaces within the parentheses.

**Fig. 9:**
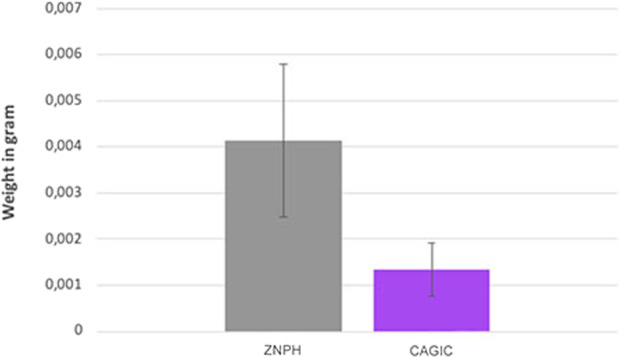
Results of weighing. Mean weight of remaining cement excess for ZNPH and CAGIC, with 95% confidence interval.

The results are presented as the mean of total number of pixels of remaining cement excess and the mean of total amount of remaining cement excess in grams on each specimen and dental cast (Figs. 8 and 9). The independent Samples *t*-Test revealed a statistically significant difference, where ZNPH had a greater amount of remaining cement excess than CAGIC, in terms of mean of total number of pixels (*p* value = 0.002) (Fig. 8) and mean of total amount in grams (*p* value = 0.005) (Fig. 9). For ZNPH, the greatest amount of remaining cement was found mesially, representing the cement excess in putty mold 6 (P6). For CAGIC, the greatest amount of remaining cement excess found distally, representing the cement excess in P2 (Fig. 8).

## Discussion

The hypothesis was accepted as a statistically significant difference in amount of remaining cement excess between the groups was observed. These differences are most likely explained by differences in mechanical properties between the two types of cement where e.g., the reduced setting time and increased hardness of CAGIC could be beneficial and facilitate removal. The brittle properties of zinc phosphate cement were thought to be favorable as the cement does not adhere to the circumjacent surfaces.^17^ However, it also led to ZNPH cement breaking down into several pieces during removal. CAGIC showed a different consistency compared to ZNPH and the removal process of CAGIC was simpler and more effective. The timing of cement removal could also have influenced the results as the cements were removed at different times during their setting process. The recommended timing of removal differs between cements. CAGIC was removed at an earlier stage than ZNPH, at a point where it reached a rubber-like consistency and could be removed as one entity. Similar findings regarding the removal process of CAGIC, were observed in Jefferies et al.^18^ Several different cements are used to cement implant-supported restorations. In the present study CAGIC was chosen for testing as it has properties that suggest easier removal. As ZNPH cement is a well-tried conventional water-based cement that has been called the “gold standard”, it was chosen as control. Cements such as resin and glass ionomer are also removed at an earlier stage during their setting process, in order to prevent it from adhering to surrounding surfaces.^17^ At this stage, the cements have obtained some strength, enough for larger pieces to be removed easily.^17^ The removal process of resin and glass ionomer cement resembles that of CAGICs. For this reason, it could be of interest to compare the different materials consistency during the removal process and amount of remaining cement excess in future studies.

After specimens were removed from the dental casts, it was observed that neither cement was completely removed. This finding is in agreement with a similar study.^13^ In the present study, the finish line was placed ~1.5 mm below the mucosal margin. This is likely to be the main explanatory factor as Linkevicius et al.^13^ found significant amounts of cement excess at submucosal margins despite operators considering that all the cement was eliminated.^13^ In the present study, more cement excess remained mesially and distally rather than buccally and palatally, in both cement types. This could be due to the proximity of neighboring teeth which made the removal process of cement excess in the interproximal areas specifically challenging. Similar results were found in a study by Lee et al.^19^ The gingival mask was perceived to have a snugger fit on the mesial side compared to the distal side. This, in combination with the consistency of ZNPH, could possibly be the reason why the cement showed significantly more remaining cement excess mesially. Apart from the snugger fit, half the papilla was made of gingival mask while the other half was made of dental stone, which made it more difficult to remove cement excess in interproximal areas. This may be considered a limitation. If the whole papilla had been made of gingival mask, it could have had better resemblance to the clinical situation.

In the present study, dental floss was used as a complement to dental probe during the removal process. These instruments are often used in the clinical situation to facilitate the removal process. Another way to eliminate cement excess is with a scaler. This technique was used in another study but did not lead to complete removal of cement remnants.^20^ Future studies need to apply and evaluate other techniques.

When the specimens were removed for pixel area calculation, it was discovered that some remaining cement excess was left in the gingival masks instead of the specimens’ surfaces. The cement that remained in the gingival mask was not included in the pixel area calculation, which thus did not represent the total amount of the remaining cement excess. Weighing on the other hand included the cement excess from both the specimen and the gingival mask, which gave a more representative result of the total amount of remaining cement excess but did not reveal if more remaining cement excess was left in the interproximal areas. This could have been illustrated for example by taking a picture of the gingival mask before the removal and weighing of remaining cement excess. However, the choice of method of establishing the total amount of remaining cement excess did not affect the outcome of the study as ZNPH cement showed significantly more remaining cement excess in both measurements.

When measuring the pixel area, six measuring areas were chosen instead of four (mesial, buccal, distal, palatal/lingual) which were used in previous studies.^13,19^ A problem that can arise when using only four measurement areas is that part of the same area is calculated twice. For example, cement excess measured on the distal side of the abutment could also be seen from the buccal side and can therefore be measured twice. This problem can be avoided when using six measurement areas, as in the present study. However, this method also has sources of error, since the measurement lines are straight, and the abutment shape is conical. This led to small areas of the abutment being left unmeasured. Therefore, guiding lines could have been drawn on the specimen before cementation to avoid measuring areas twice or leaving areas unmeasured. However, this is not likely to have affected the outcome of the present study as this limitation would affect both groups in a similar way.

## Conclusion

Within the limitations of this in vitro study, the findings suggest that the amount of remaining cement excess can be affected by the type of cement and that CAGIC leaves less remaining cement excess compared to ZNPH cement. CAGIC may thus be a more suitable choice for cement-retained dental implant restorations and could possibly lead to reduced biological peri-implant complications. This latter hypothesis must however be tested in further clinical studies.
